# Smart, Remote, and Targeted Health Care Facilitation Through Connected Health: Qualitative Study

**DOI:** 10.2196/14201

**Published:** 2020-04-28

**Authors:** Sonia Chien-I Chen, Ridong Hu, Rodney McAdam

**Affiliations:** 1 Institute of Quantitative Economics Huaqiao University Xiamen China; 2 Ulster Business School Ulster University Newtownabbey United Kingdom

**Keywords:** connected health care, smart health care, health care quality, access, remote monitoring, precision medicine, self-management

## Abstract

**Background:**

Societies around the world are aging. Widespread aging creates problems for social services and health care practices. In this light, research on connected health (CH) is becoming essential. CH refers to a variety of technological measures that allow health care to be provided remotely with the aim of increasing efficiency, cost-effectiveness, and satisfaction on the part of health care recipients. CH is reshaping health care’s direction to be more proactive, more preventive, and more precisely targeted and, thus, more effective. CH has been demonstrated to have great value in managing and preventing chronic diseases, which create huge burdens on health care and social services. In short, CH provides promising solutions to diseases and social challenges associated with aging populations. However, there are many barriers that need to be overcome before CH can be successfully and widely implemented.

**Objective:**

The research question of this study is as follows: How can CH facilitate smart, remote, and targeted health care? The objective is to identify how health care can be managed in more comprehensive ways, such as by providing timely, flexible, accessible, and personalized services to preserve continuity and offer high-quality seamless health care.

**Methods:**

A qualitative approach was used based on 60 multistage, semistructured stakeholder interviews.

**Results:**

The results can be divided into two functions of CH: ecosystem and platform. On the one hand, the interviews enabled the authors to develop a stakeholder classification and interaction diagram. These stakeholders interacted sequentially to provide technology-based content to end users. On the other hand, interviewees reflected on how CH serves as a platform to address remote monitoring and patient self-management. In the Discussion section, three innovation strategies are discussed to reflect the manner in which CH promotes smart, timely, and precise health care.

**Conclusions:**

This study indicates that it is essential to continually revise CH business models, given the ongoing and rapid changes in technology across groups of CH stakeholders. We also found that global trends toward smart, timely, and precise health care shape what individuals expect from products and services, providing firms with unique opportunities for growth.

## Introduction

### Background

Our society is aging. This trend is projected to result in increased chronic health conditions and potential labor shortages. As traditional health care models are not fully equipped to face the unique challenges of providing health care for an aging society, innovative practices are being proposed and developed to decrease the pressures of widespread aging. Through self-management and remote monitoring platforms, connected health (CH) has been offered as a new technology-based model of health care delivery that presents a promising solution for future, aging-oriented health care [1].

The rise of CH is attributable to four factors. The first factor is related to the widespread tendency to pursue excellence in health care through the promotion and monitoring of health care services’ quality, efficiency, safety, and level of customer service. Because of this tendency, health professionals and institutions demand that health care services be more accessible, of a higher quality, and more efficient, so they often turn to CH [2]. Second, aging populations have increased health care costs due to the increase in chronic conditions, higher survival rates among patients fighting serious diseases, and longer lifespans that accompany aging [3]. Thus, the health care economy has become more complex than in the past as evidenced by the rising costs of caring for changing demographics. Third, increasingly frequent provider shortages and other relevant issues, including the geographic dispersion of families and troubling disparities in care between ethnic groups, are significant concerns for aging populations [4]. Lastly, patients in varying contexts have demanded better customer service from health care providers [5]. Some authors suggest that the development of consumerism in health care may be a catalyst for the development of patient-centric health care [6-8]. In combination, these factors have created a stronger impetus to force health care innovation from both within and outside the ecosystem.

Although CH seeks to make health care services more proactive, preventive, and precisely targeted, there are many challenges and barriers to its sustainable implementation that need to be addressed and overcome. These challenges include the cost of devices, privacy and data security concerns, health system bureaucracy, and training health professionals and patients to use new technologies. This study identifies strategies to make health care systems smarter, timelier, and more precise by exploring the interaction of stakeholders in the CH ecosystem and CH’s role as a platform.

### Study Design and Conceptual Framework

**Figure 1:**
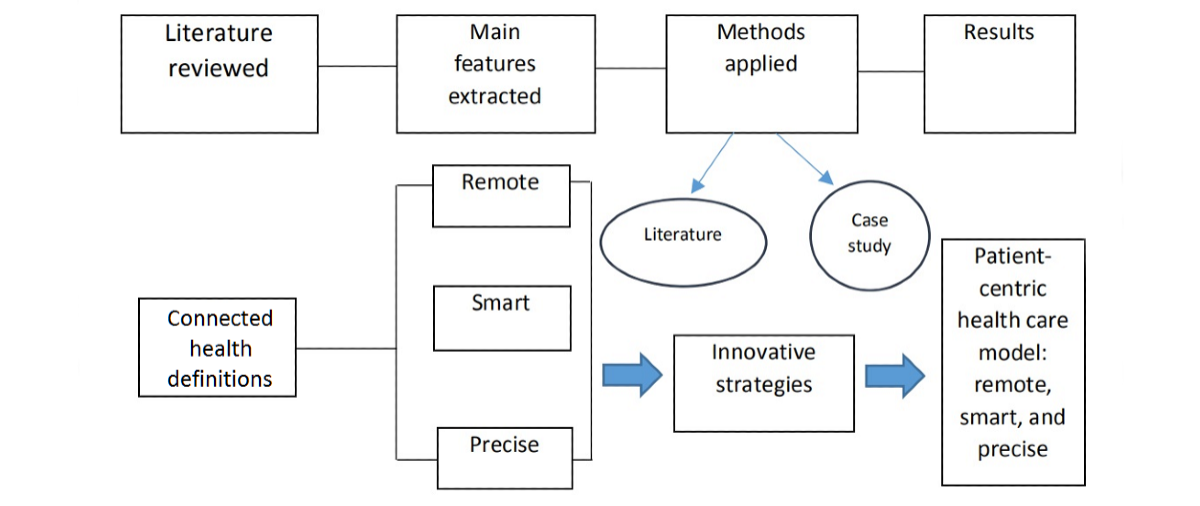
The study’s design and conceptual framework.

Figure 1 illustrates the conceptual framework that forms the basis for this study. First, we reviewed definitions of CH in the literature to determine its main features. Second, three features of timely, smart, and targeted care were determined and applied to evaluate how patient-centric health care services can be delivered through CH. Third, we conducted interviews and engaged case studies to identify effective strategies for the implementation of CH. Although CH is promising, many challenges, such as issues of cost, infrastructure, technology, and business sustainability, still remain to be overcome. Therefore, we examined extant and innovative strategies for implementing CH and/or augmenting existing health care systems and interviewed relevant stakeholders in the CH ecosystem to discover how to reach these goals. Finally, the results were analyzed with an eye to expressing the implications of addressing a comprehensive health care system that can fulfill patients’ needs and suggestions on how to do so.

### Definition of Connected Health

Broadly speaking, CH is an *umbrella term* that covers the telemedicine family spectrum. An increasingly developed base of knowledge around CH and CH practices has generated many definitions of exactly what CH is [2,8]. For instance, the American Medical Association (AMA) defines CH as a model that utilizes technology to maximize health care resources and offer enhanced, flexible opportunities for patients to engage with clinicians and better self-manage their care [9]. In order to achieve these goals, many technologies, including telemedicine and mobile health, are used to facilitate remote, mobile, and site-to-site medical care. To focus our research, we classified definitions of CH into three types based on their salient features: remote, smart, and precise. This study then defines CH as a platform that offers remote monitoring and self-management, helping patients to address their own health care needs in a smart, timely, and precise manner through information and communication technologies (ICTs). Below, we briefly discuss our three classifications of CH definitions.

### Remote Health Care

Remote medical care is a key feature of CH [3-5,9-12]. Studies indicate that both the concept of remote health care and the practical application of information technology systems to health care include aspects of remote medical care, such as a *telecare medicine information system*, a *personally controlled health records system*, and *patient monitoring*. These studies further point out that in CH applications, “user authentication can ensure the legality of patients’ care” [13]. Remote health care–focused definitions of CH are often seen as part of a new lexicon for telemedicine, even though these definitions and their proponents may exhibit a greater focus on how to best connect clients and health care professionals.

### Smart Health Care

Although technology plays a significant role in CH, researchers indicate that CH is not just about technologies, but its relationship with people and the health care system [14]. Smart-oriented definitions of CH demonstrate how CH is not just about health care and technology, but about managing patients and their care [12,14]; they indicate that *CH is a new model for health management* and that CH has the potential to *put the correct information in the correct hands at the correct time*, thus improving decision making and care. These decisions can “save lives, save money and ensure a better quality of life during and after treatment.” CH can help deliver better health outcomes by allocating health resources more efficiently and effectively via the better management and integration of health care systems and services, which are, in theory, typified by smart health care.

According to the literature review, CH tends to be a combination of people, processes, and technology [4,15]. Take, for example, the following definition: “connected health...refers to a conceptual model for health management where devices, services or interventions are designed around the patient’s needs” [15]. Based on this definition, CH is a patient-centered care model where the patient is at the center of the processes that connect stakeholders—a process that takes place over a variety of settings, from the patient’s home to an acute care setting. This definition also indicates that patient care pathways, revenue models, and data analytics can be connected through technology in the CH platform. Therefore, technology can enable the implementation of a more proactive, episodic health care model, which is starkly different from the more reactive model of conventional health care. With the assistance of technology, health care professionals, patients, and/or caregivers can be empowered to engage in more effective and efficient health care.

### Precise Health Care

CH differs from conventional health care in that it collects data in a timely and seamless fashion, thus providing more information for clinicians to address patients’ precise health care needs [2]. Furthermore, “stakeholders in the process are connected...using timely sharing and presentation of accurate and pertinent information regarding patient status through the smarter use of data, devices, communication platforms and people” [16]. Therefore, through CH, patients can receive care in a manner that is as efficient and precise as possible.

### Value Propositions Based on Trends

As noted above, the patient-centric values of CH can be defined and classified into smart, timely, and precise care. The relevant literature reviews how such values can be proposed and captured through strategies. Values are created by a value chain comprising suppliers, firms, and buyers [17]. How much value can be captured via strategies depends on the capacity of these strategies to add value to a given service or care practice. The analysis of value‐based business strategies depends on the cooperative game theory among firms, how they act toward one another as competitors, firms’ willingness to pay, and the opportunity costs of acting. Although value types vary, value propositions in business models are the key to increase the attraction of firms’ willingness to pay [18]. Three innovative strategies have been identified to potentially rebuild the business model: *infuse and augment*, *combine and transcend*, and *counteract and reaffirm* [19]. We review examples from prominent companies in the literature to support the idea that these strategies can influence success in service innovation [12,19]. Furthermore, innovation should be inspired by clients’ motivations and innovations’ appeal to clients; therefore, using these strategies may be an effective way to achieve this goal [20].

Despite the importance of value propositions and strategic planning, most research focuses on two primary performance factors: cost-effectiveness and operational efficiency [21]. Although the qualities of timely, smart, and precise health care are essential to create a patient-centric health care model, more research is required to explore how these goals can be achieved in practice. According to the literature, technology is not the sole factor driving more personalized health care services. Management and other factors that involve qualitative components may help to maintain human esteem and dignity through a CH platform [22,23]. Although CH’s value has been demonstrated in the management of chronic diseases, researchers expect tremendous progress in CH’s applications in disease prevention [24]. The proliferation of advanced technology enables CH to be more proactive than conventional medicine [25]. Thus, a qualitative approach is employed to achieve the goal of providing more proactive, preventative, and precise health care.

Despite the positive impacts of CH described above, barriers to implementing CH still need to be identified and overcome in order to accelerate and deliver more effective and cost-efficient health care models [15]. Health care that is proactive, preventive, and targeted is likely to reduce many of the burdens of an aging society [26,27]. Yet, there is still a gap in the research regarding how medical interventions such as the implementation of CH can both be effective and also maintain the dignity of elderly patients [28]. To fill this gap, this study explores the relationship between technology and wellness, and how both have been optimized in certain cases. It identifies the preconditions and essential requirements for developing a CH ecosystem and provides typical, illustrative case studies to answer the research question. Therefore, this study aims to qualitatively explore and discover hitherto unknown aspects of how health care can be more comprehensive by being smarter, timelier, and more precise.

## Methods

### Overview

This study uses literature reviews and empirical research to investigate how timely, smart, and targeted health care can be achieved via CH platforms. Although the majority of CH research has used quantitative methods to test and validate assumptions [29-31], this study utilized a qualitative research method, conducting 60 interviews that were designed to answer the research question and explore the unknown and novel phenomena of health care in an aging society. We employed three methods to identify stakeholders in the CH ecosystem: (1) reviewing the CH organizational process assets (ie, the plans, processes, policies, procedures, and knowledge bases specific to and used by the performing organization) and their environmental factors; (2) interviewing experts in the field of CH; and (3) conducting brainstorming sessions. Regarding CH-related value propositions, the strategies based on trends in the literature are relevant. Therefore, the innovative strategies proposed in previous studies [19] are employed as a foundation for classifying and discussing the results of this study.

### Research Methodology

Owing to the developmental nature of CH literature, this study used an exploratory and inductive theory-building approach based on our 60 multistage, semistructured stakeholder interviews. The target sample we used to address the study aims and research question was CH stakeholders and experts, including medical researchers, industry leaders, government officials, and end users. This qualitative methodology facilitates a better understanding of a rapidly developing discourse, such as that of CH, by exploring and using insightful input, having broader discussions, and synthesizing diverse opinions [29,31]. Thus, this approach can be used to bridge the knowledge gaps in literature.

### Recruitment Criteria of the Participants

Stakeholders and experts were selected for the interviews according to the literature review and snowball sampling [32]. These participants were interviewed on a voluntary basis and gave verbal consent according to ethical guidelines. This study was approved by Ulster University’s institutional review board (reference No. RG3 RMcAdam2). The study’s participants included health care professionals, industry players, academic researchers, and government agents. The researchers identified participants’ backgrounds, competencies, gender, etc, as they may relate to the outcomes of this research. Our semistructured interview guide comprised the following: (1) working sectors, (2) background information, (3) gender, and (4) levels of competency.

Taiwan was selected as a case study to explore the myriad phenomena accompanying aging populations and test strategies for the development and implementation of CH. We chose this case study because Taiwan has experienced both significant challenges and progress in its core health care activities. Another reason for choosing Taiwan was because of its complete CH business ecosystem, which includes mature ICT applied to health care practices and an integrated health care system. Approaching our research questions through the multi-stakeholder case study of Taiwan helps us to explore rich contextual information by asking *what* and *how* questions, rather than generalizing principles to a population [29,31]. Multistage interviews were conducted to deepen the research by iteratively collecting data [29]. The data were collected using a multistage approach with interviewees, considering the CH ecosystem’s progressive and multidimensional nature. This is illustrated in Tables 1-3.

Pilot interviews were conducted (n=16) with key CH influencers across stakeholder groupings to explore the CH phenomenon and identify the primary contributing factors and stakeholders in Taiwan’s CH ecosystem. Next, Stage 1 interviews (n=22) were held with CH stakeholders who were identified from the pilot stage interviews’ findings and analysis. An analysis was conducted using a snowball sampling method [32]. Stage 2 interviews (n=22) were conducted 1 year after the Stage 1 interviews to explore how systemic problems develop and persist over time in CH ecosystems and to review longitudinal changes from a Business Model Innovation (BMI) perspective in terms of the sustainability of CH.

The pilot interviews were conducted in order to confirm the need for, and significance of, further CH research. This pilot study was also advantageous because it identified potential directions and perspectives for conducting the multistage interviews, which helped us collect data more effectively. The results of the pilot interviews suggested that further study of CH is meaningful. Barriers that obscured the development of CH were further investigated in the Stage 1 interviews. As mentioned above, 16 interviewees were included in the pilot interviews (see Table 1). The majority were health care providers, as they have rich experience of CH technologies and services. Their feedback was significant in reflecting the effectiveness of CH products and services. Therefore, understanding their views is important in the design of successful and user-friendly interactive systems. This pilot interview focused on medical doctors and health professionals rather than patients and their families, due to ethical concerns.

**Table 1:** Profiles of the pilot study interviewees.

Participant number	Sector and participants in that sector (n=16), n (%)	Organization	Gender^a^	Title
1	Industry, 2 (13)	June Sun Digicom	Male	General Manager
2	Industry, 2 (13)	DigiO2	Male	Tech Advisor
3	Government or semigovernment, 2 (13)	Taiwan Forces for Medical Travel	Male	Chief Executive Officer (CEO)
4	Government or semigovernment, 2 (13)	Taiwan Forces for Medical Travel	Female	Vice Project Manager
5	Academia, 1 (6)	Taipei Medical University, School of Gerontology Health Management, College of Nursing	Female	Assistant Professor or Attending Physician
6	Health care providers, 11 (69)	Chang Gung Memorial Health Village	Female	Staff
7	Health care providers, 11 (69)	Antai Medical Care Hospital	Male	Director or General Practitioner (GP)
8	Health care providers, 11 (69)	Antai Medical Care Hospital	Female	GP
9	Health care providers, 11 (69)	San-Chung Health Center	Female	Head Nurse
10	Health care providers, 11 (69)	YR Chinese Medicine Clinic	Male	Doctor
11	Health care providers, 11 (69)	YR Chinese Medicine Clinic	Female	Manager
12	Health care providers, 11 (69)	Tri-service General Hospital	Male	Medical Doctor
13	Health care providers, 11 (69)	Taiwan University Hospital	Male	Medical Doctor
14	Health care providers, 11 (69)	Kaohsiung Municipal Hsiaokang Hospital	Female	Pharmacist
15	Health care providers, 11 (69)	Zhang Bi Zheng Family Physicians’ Clinic	Male	Director or GP
16	Health care providers, 11 (69)	Home Physician	Male	Therapist

^a^The sample was made up of 56% (9/16) males and 44% (7/16) females.

**Table 2:** Profiles of Stage 1 interviewees.

Participant number	Sector and participants in that sector (n=22), n (%)	Organization	Gender^a^	Title
17	Industry, 8 (36)	Netown	Male	Sales
18	Industry, 8 (36)	Far EasTone Telecommunications	Male	Senior Engineer
19	Industry, 8 (36)	Far EasTone Telecommunications	Male	Senior Engineer
20	Industry, 8 (36)	Huede Technology	Male	Chief Executive Officer (CEO)
21	Industry, 8 (36)	Huede Technology	Female	Nurse or Health Management
22	Industry, 8 (36)	Guidercare	Male	Vice President
23	Industry, 8 (36)	Acomotech	Male	Tech Advisor
24	Industry, 8 (36)	Medsense	Male	Founder
25	Government, 2 (9)	Ministry of Health & Welfare	Male	Director of ICT^b^ Department
26	Government, 2 (9)	Sang Chung Health Center	Male	Administrator
27	Academia, 3 (14)	National Taiwan University	Male	Director or Professor
28	Academia, 3 (14)	National Taipei University of Technology	Female	Lecturer
29	Academia, 3 (14)	UL (Underwriters Laboratories) Life & Health	Male	Sales Manager
30	Health care providers, 9 (41)	Luo Dong Care Institute	Male	CEO
31	Health care providers, 9 (41)	En Chu Kong Hospital	Female	Senior Nurse
32	Health care providers, 9 (41)	Taoyuan Fu Hsing Township Health Station	Male	Director or General Practitioner (GP)
33	Health care providers, 9 (41)	Taoyuan Fu Hsing Township Health Station	Female	Head Nurse
34	Health care providers, 9 (41)	Taipei Medical University, Telehealth & Telecare Center	Female	Director or Health Management
35	Health care providers, 9 (41)	Mennonite Christian Hospital	Male	Management of Information Service (MIS) Director
36	Health care providers, 9 (41)	Mennonite Christian Hospital	Female	Head Nurse
37	Health care providers, 9 (41)	Taiwan University Hospital, Telehealth Center	Female	Nurse or Health Management
38	Health care providers, 9 (41)	Taiwan University Hospital	Male	GP

^a^The sample was made up of 68% (15/22) males and 32% (7/22) females.

^b^ICT: information and communication technology.

**Table 3:** Profiles of Stage 2 interviewees.

Participant number	Sector and participants in that sector (n=22), n (%)	Organization	Gender^a^	Title
39	Industry, 15 (68)	Agfa HealthCare Image (imaging technology)	Male	Consultant
40	Industry, 15 (68)	Asus Compute Inc (internet technology)	Male	Senior Manager
41	Industry, 15 (68)	G-cloud UK (cloud computing technology)	Male	Chief Executive Officer (CEO)
42	Industry, 15 (68)	Sheng-En Development Co (value network)	Male	Division Manager
43	Industry, 15 (68)	Asus Cloud Corporation (internet, storage, and cloud computing technology)	Male	Department Director
44	Industry, 15 (68)	Far EasTone Telecommunications	Male	Director
45	Industry, 15 (68)	Taidoc/Fora Care	Male	Sales Manager
46	Industry, 15 (68)	Smart Catch International Co, Ltd	Female	Specialist
47	Industry, 15 (68)	MitraStar	Male	Senior Director
48	Industry, 15 (68)	Gemtek Technology Co, Ltd	Female	Product Manager
49	Industry, 15 (68)	Z-Com/ZWA Inc	Male	Sales Director
50	Industry, 15 (68)	Isentek/Partnership with TXCorpration	Female	Deputy Sales Manager
51	Industry, 15 (68)	Through Tek Technology (TUTK) Co, Ltd	Female	Product Manager
52	Industry, 15 (68)	Pioneer Material Precision Tech	Female	Buyer
53	Industry, 15 (68)	Auden Group	Female	Executive Assistant
54	Health care providers, 6 (27)	CH^b^ Healthy Village (value network)	Female	Division Manager
55	Health care providers, 6 (27)	Changhua Christian Hospital (CCH) (telecare health service center) (BMI^c^)	Female	Head Nurse
56	Health care providers, 6 (27)	CK Memorial Hospital (health professional)	Female	Senior Pharmacist
57	Health care providers, 6 (27)	Tai Tong Health Center (CH consumer in remote area)	Male	Medical Doctor
58	Health care providers, 6 (27)	Show-Chwan Hospital	Female	Nurse
59	Health care providers, 6 (27)	Show-Chwan Hospital	Female	Nurse
60	Academia, 1 (5)	Industrial Technology Research Institute/Ministry of Economic Affairs (MOEA)	Female	Manager

^a^The sample was made up of 45% (10/22) males and 55% (12/22) females.

^b^CH: connected health.

^c^BMI: Business Model Innovation.

The Stage 1 interview asked participants to consider problems reported in the literature on CH. These questions covered the cost of implementing CH and creating the necessary infrastructure and technology, business sustainability, different CH business models, collaboration, and communication-related issues. The results were analyzed to identify further knowledge gaps and to increase the depth of current knowledge. Stage 2 interviews addressed the gaps identified in Stage 1 and sought to discover insights of sustaining CH businesses. Due to the challenges of time limits and the distance of remote areas in Taiwan during data collection, we required two phases of interviews to ensure that we adequately covered some important issues and themes.

Interviews lasted between 1 and 2 hours and were transcribed and coded using NVivo 10 (QSR International) qualitative data analysis software. Different data sources, including interviews, documents, and public records, were synthesized and coded for data triangulation to increase the credibility and validity of the study’s results [29]. Data were cleaned through an integration process to merge different terms with similar meanings. For example, CH could be called *remote health*, *telehealth*, and *telecare* in the interviews; these terms were merged because the interviewees used them to refer to the same thing. Inaccurate, incomplete, or irrelevant parts of the dataset were detected, corrected, or removed from the raw data in the data-cleaning phase. The data were then clustered and coded.

The data analysis strategy of this study included thematic and systematic approaches in a collaborative qualitative analysis. The data collected underwent a deep familiarization, and this led to the development of major themes, which in turn organized the thematic analysis. This study utilized NVivo 10 to analyze the interview data by grouping words or sentences based on how frequently they occurred in the interviews. Content analysis was conducted to objectively and systematically induce meaning on the content through coding [33]. This analysis adopted a grounded theoretical approach. Initially, we drew out coded data and themes with the potential for rapid change. The number of new and emerging themes gradually diminished, and the number of refinements to these themes decreased until theoretical saturation was met. The data collection and iterative analysis were repeated until saturation occurred. The data were summarized, confirmed, and discussed in subsequent interviews with the same interviewees from the pilot stage, Stage 1, and Stage 2. This process was intended to ensure the data’s reliability and validity. This method is beneficial as the interviews’ primary content can be confirmed and corrected quickly by experts in the CH ecosystem. Throughout the process, constant reference to existing literature contributed to the data analysis [29].

## Results

This study employed both thematic analysis and systematic content analysis. The results are sequentially demonstrated in the following subsections.

### Results of Thematic Analysis

**Figure 2:**
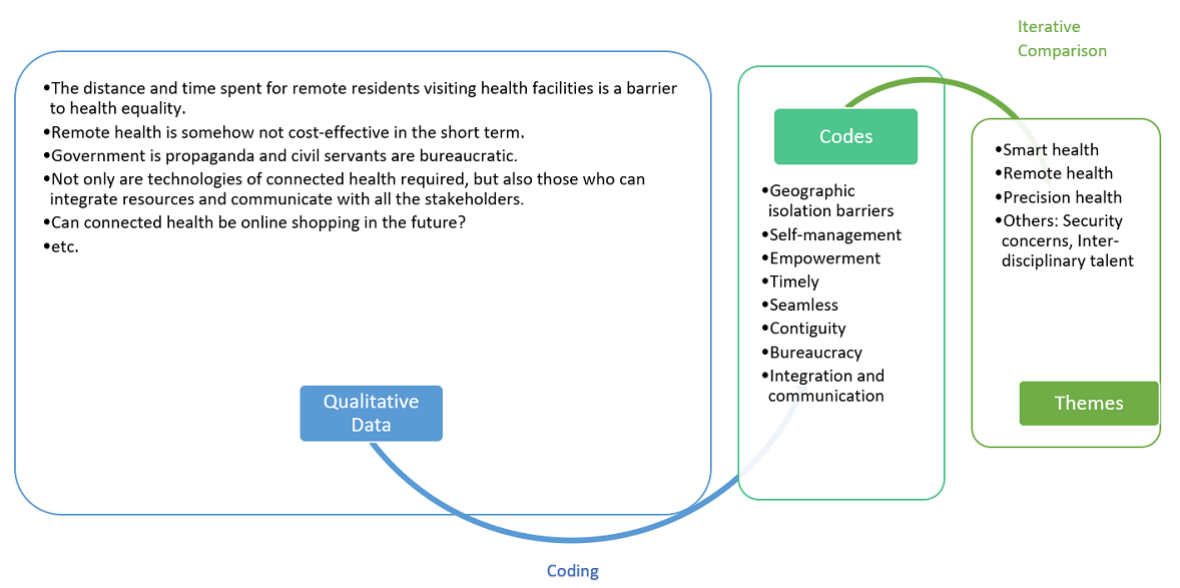
Results of thematic analysis.

Figure 2 shows the results of thematic analysis in terms of how qualitative data were coded and iteratively compared and integrated into themes. For instance, the description “the distance and time spent for remote residents visiting health facilities...” and the non-cost-effectiveness of “...remote health...” were coded as geographic isolation barriers and developed into the theme of *remote health* according to iterative comparison. Other interview data were clustered and merged according to a similar mechanism.

**Figure 3:**
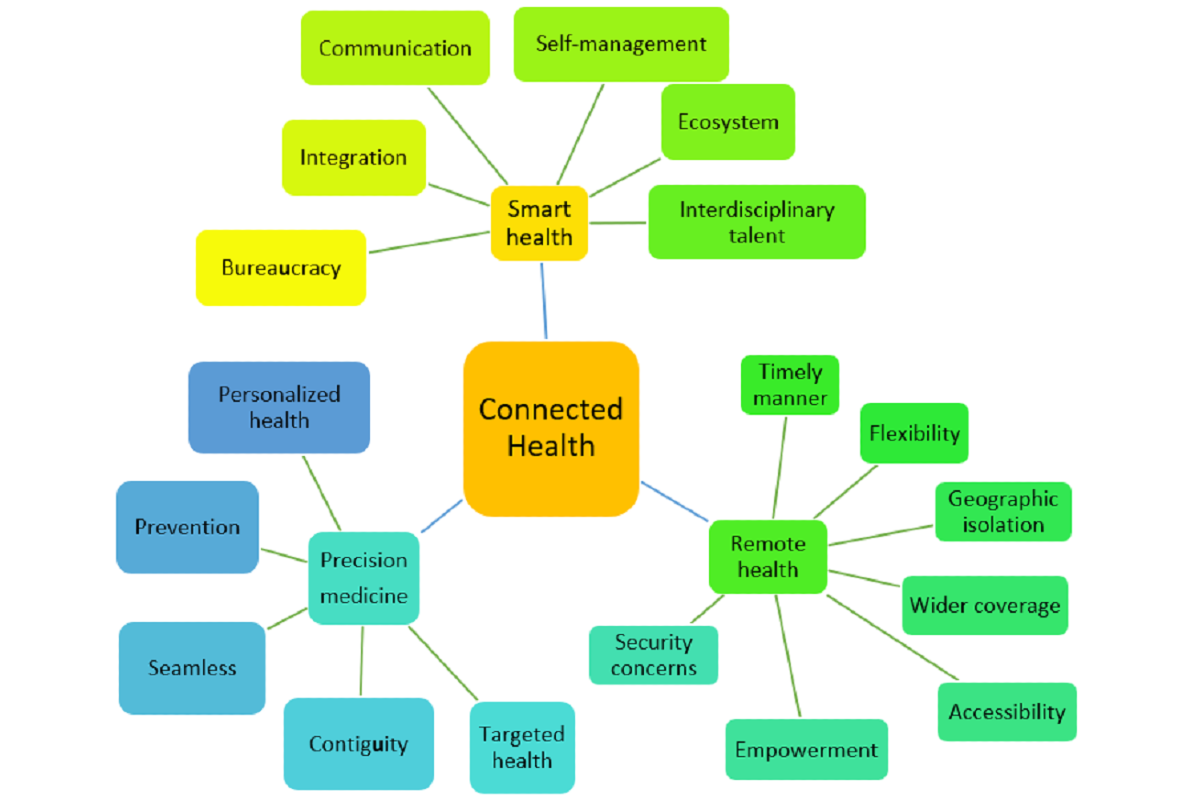
Thematic analysis mapping.

Figure 3 illustrates a map of how codes converged as themes from the qualitative data. Codes for integration, communication, bureaucracy, ecosystem, self-management, and interdisciplinary talent were merged as the theme *smart health*. Seamless, contiguity, prevention, targeted health, and personalized health were categorized into *precision medicine*. The theme *remote health* covered flexibility, accessibility, empowerment, timely manner, geographic isolation, wider coverage, and security concerns. In summary, smart health, precision medicine, and remote health consisted of the CH features.

### Results of Systematic Analysis

NVivo 10 was used to analyze the interview data by grouping words or sentences based on how frequently they occurred in the interviews. The results are shown in Table 4 and Multimedia Appendix 1. According to the word cloud of Multimedia Appendix 1 it is noticeable that keywords such as care, business, data, system, model, time, technology, government, and management were most frequently mentioned in the interviews. The word-frequency framework was used to identify meaningful words and their potential significance. The words that frequently occurred were clustered into three themes: technology, organization, and leadership and management issues (see Table 4). These words that were shown the most frequently were compared according to the original context and grouped into key themes.

**Figure 4:**
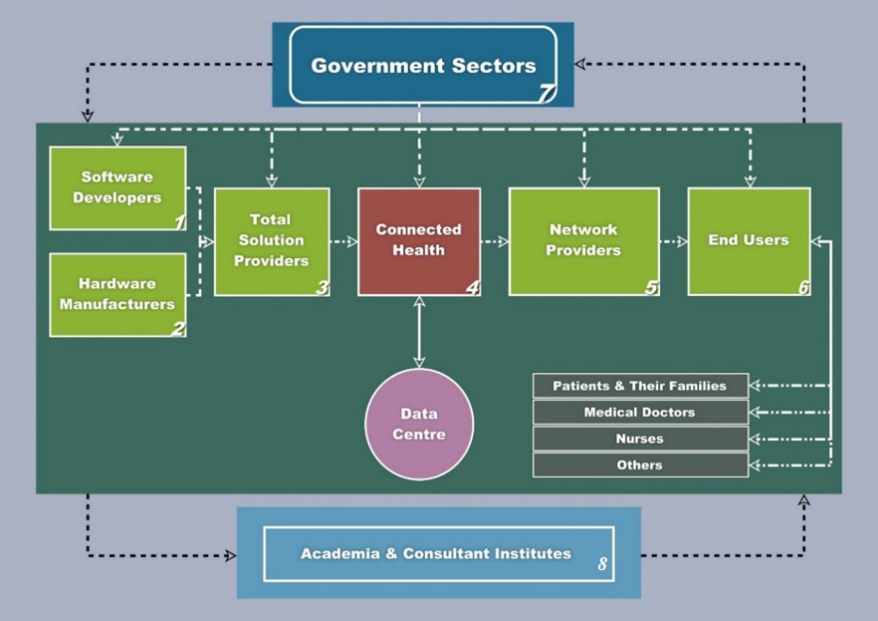
Diagram of connected health players and stakeholder relationships (Chen, 2018 [34]).

Results of the analyses conducted on the interview data are summarized and presented in the following sections: (1) CH as an ecosystem, (2) CH as a platform, and (3) CH’s innovative strategies based on value propositions. First, the principles adopted to identify stakeholders in the ecosystem, based on the power and interest of their ability to affect CH’s objectives, were reviewed. The stakeholder interaction diagram (see Figure 4) was created based on the foundation of a previous study that identified stakeholders in the CH ecosystem [34]. In our diagram, governments take the lead and initiate the infrastructure projects needed to implement CH, and the sequential interaction that follows provides technology-based content to end-user stakeholders. Second, CH as a platform enables remote monitoring and makes patients’ self-management possible. Moreover, interviewees contributed insights regarding the challenges that the implementation of CH faces and how they can be addressed. Third, CH’s innovative strategies based on value propositions were referred to the value propositions from current trends, which may inspire practitioners and policy makers. Three innovation strategies, based on information emanating from Harvard Business School, are discussed to reflect how timely, smart, and targeted health care can be delivered via the CH platform [12].

Figure 4 illustrates the overall CH ecosystem, along with relationships between stakeholders and players [35]. Figure 4 identifies eight types of stakeholders and players, which can be classified into three sectors: government, industry, and academia. Policy making and infrastructure building, led by government, initiates the implementation of CH. Figure 4 demonstrates that governments are influential throughout the CH stakeholder ecosystem, as they fund many short-term projects and initiate the infrastructure required for CH’s implementation.

Academia allows the research, discovery, innovation, and transfer of knowledge regarding CH processes to entities such as government stakeholders, and academic stakeholders are influential throughout the CH ecosystem. Although academia is primarily involved in the development of CH technology, it has become increasingly involved in the social, business, and marketing aspects of value-centric CH.

Finally, industry stakeholders are major players in the CH ecosystem who offer services and manufacturing products for use by end users. Industry covers a variety of domains: (1) software developers, (2) hardware manufacturers, (3) total solution providers, (4) CH service providers, (5) network services, and (6) end-user stakeholders. Besides the three major stakeholders mentioned above, other actors in the CH system include patients, patients’ families, doctors, nurses, caregivers, and additional persons, primarily volunteers.

**Table 4:** Themes and word frequencies.

Theme and words		Word frequency (N=61,489), n (%)
**Technology issues**		
	Care	289 (0.47)
	Data	152 (0.25)
	System	116 (0.19)
	Model	112 (0.18)
	Technology	109 (0.18)
	Time	108 (0.18)
	Information	93 (0.15)
	Remote	79 (0.13)
	Distance	72 (0.12)
	Concept	64 (0.10)
	Support	64 (0.10)
	Research	61 (0.10)
	Staff	61 (0.10)
	Personal	60 (0.10)
	Policy	60 (0.10)
	Measuring	57 (0.09)
	Cost	53 (0.09)
	Innovation	53 (0.09)
	Quality	48 (0.08)
	Mobile	40 (0.07)
	Cloud	42 (0.07)
	Device	39 (0.06)
	Smart	34 (0.06)
**Organization issues**		
	Government	95 (0.15)
	System	116 (0.19)
	Business	189 (0.31)
	Model	112 (0.18)
	Support	64 (0.10)
	Concept	64 (0.10)
	Staff	61 (0.10)
	Public	68 (0.11)
**Leadership and management issues**		
	Management	83 (0.14)
	Support	64 (0.10)
	Time	108 (0.18)
	Research	61 (0.10)
	Concept	64 (0.10)

### Connected Health as a Coexistent Ecosystem

Smart health care via a CH platform explores how health care and technology can coexist, each dependent on the other, rather than operating as separate entities in the CH ecosystem. This coexistence not only creates a health care system that can better respond to the needs of an aging society, but has the side effect of developing smarter health care methods. Therefore, it is relevant to reconsider the conventional concepts of technology and health care. As an ecosystem, CH is surrounded by software providers, hardware manufacturers, and health care professionals (see Figure 4).

As far as the sensitive nature of health care data is concerned, cybersecurity plays an important role in ensuring the safety of patients’ data. Therefore, it is critical to include the role of *information technology (IT) security specialists* in the CH ecosystem. They are incorporated within both the categories *software developers* and *hardware manufacturers*. Other relevant roles are covered in these eight categories, even though not all of them are named in Figure 4. For example, the role *stakeholders with standardization* is included within the category *academia and consultant institutes*. What is noticeable is that the role of *interoperability personnel* still remains to be developed according to many cases interviewed, although this role is essential for the success of CH’s implementation.

Followed by the main stakeholders in Figure 4, the classification of organizations and participants interviewed is provided in Table 5. This table classified the 60 participants from 34 organizations into eight categories according to the categories in Figure 4. When we found that some organizations bridged two or three categories, we classified them according to their main businesses. Despite having their own businesses and business concerns, they have to coexist to activate CH’s implementation.

### Connected Health as a Platform of Remote Monitoring and Self-Management

This section presents the interview results regarding how CH functions as a platform of remote monitoring and self-management, based on the perspectives of participants shown in Table 6.

**Table 5:** The classification of organizations interviewed.

Category and numbers		Organization name
**1. Software developers**		
	101	Far EasTone Telecommunications Smart City Division
	102	Guidercare
**2. Hardware manufacturers**		
	201	Netown Corporation
	202	Far EasTone Telecommunications Technical Department
	203	Huede Technology
	204	Guidercare
	205	Acomotech
**3. Total solution providers**		
	301	Netown Corporation
	302	Far EasTone Telecommunications
	303	Huede
	304	Guidercare
	305	Acomotech
**4. Connected health (CH) service providers**		
	401	Luo Dong Care Institute
	402	En Chu Kong Hospital
	403	Taoyuan Fu Hsing Township Health Station
	404	Taipei Medical University, Telehealth & Telecare Center
	405	Mennonite Christian Hospital
	406	Taiwan University Hospital, Telehealth Center
	407	Taiwan University Hospital
**5. Network providers**		
	501	Far EasTone Telecommunications
**6. End users**		
	601	National Taiwan University Hospital
	602	Mennonite Christian Hospital
	603	Taipei Medical University, Telehealth & Telecare Center
	604	En Chu Kong Hospital
	605	Luo Dong Care Institute
**7. Government sectors**		
	701	Health & Welfare Department
	702	Sang Chung Health Center
	703	Luo Dong Care Institute
	704	Taoyuan Fu Hsing Township Health Station
**8. Academia**		
	801	National Taiwan University, Engineering Department
	802	National Taipei University of Technology
	803	Taipei University
	804	UL (Underwriters Laboratories) Life & Health
	805	National Taiwan University, Medicine Department

**Table 6:** Participants’ perspectives regarding how connected health (CH) functions as a platform of remote monitoring and self-management.

Participants	Summary	Quotes
1 and 7	Most interviewees indicated that remote service is a promising solution for working adults who have to balance multiple priorities in limited time and care for aging parents or dependents. In a CH ecosystem, their parents might receive services remotely, with smart and precise features tailored to their specific course of treatment. Some respondents indicated that they were willing to receive smart services if these services were available and accessible in their areas. Additionally, smart, precise, alternative health care solutions effectively reduced the restraints placed on care through the time and cost of travelling, particularly for patients who were located outside of major metropolitan areas.	“It will be beneficial if remote services are available to look after both parents and children at home. At the same time, sending older parents to a care institution is not our culture after all.” [Participant #1] “Remote solutions offer residents in rural areas efficient and cost-effective options to receive health care services.” [Participant #7]
37, 4, 15, and 20	Many interviewees agreed that, apart from alternative solutions, remote services add value to health care centers by ensuring that stakeholders and players in the CH ecosystem remain informed and connected. These services not only enable residents in less-populated areas to receive health care services, they ameliorate patients’ concerns about being isolated from essential health care services and needs.	“Our customers are connected with hospital care, thanks to the remote monitoring.” [Participant #37] “Remote services make the scalability of health care possible.” [Participant #4] “A professional exchange of opinions is enabled through remote service.” [Participant #15] “Our remote device and service enable patients to be monitored at home after their surgery...Both patients and the hospital can benefit from this facility.” [Participant #20]
43, 4, and 37	Although some industrial stakeholders believed that health care should be localized, some policy makers believed that Taiwan could attract international visitors by promoting medical tourism, which would allow a future scaling-up of health care businesses as these individuals pay for and receive comprehensive services. Whether the Taiwanese health care model and service can be expanded overseas is still debatable; however, what is certain is that the results suggest that remote services are particularly helpful for overseas businesspeople since these services offer seamless, timely, health-monitoring services. Participants made comments regarding medical tourism in the Taiwanese context (see quotes in next column).	“I think medical services should remain localized as they are developed according to local needs and culture.” [Participant #43] “Taiwan has excellent health and medical products and services; these advantages should be attractive to international tourists...It is a good opportunity for us to develop medical tourism.” [Participant #4] “Many of our customers are Taiwanese businessmen who work in mainland China...we have expanded the telehealth service from the island of Taiwan to include mainland China...” [Participant #37]
10, 14, and 13	Whether a one-size-fits-all approach to remote services is feasible is debatable. Some conventional health care providers are conservative in their implementation of remote practices. Concerns expressed included the costs of initiating investment in infrastructure and ensuring a return on those investments. Health professionals continued to disagree as to whether remote services can actually translate to more effective care or can replace personal provider-patient interactions. Although these are future trends in health care, some doctors still held more conservative views.	“Remote service is an ideal option; however, due to our business scale and direction, we are not yet offering this service.” [Participant #10] “In the short term, remote service can be a sponsored pilot program; however, a significant amount of time is needed to receive its return on investment.” [Participant #14] “After a decade of practicing medical service, I personally believe health care should be implemented face-to face...” [Participant #13]
7	The stakeholders that we interviewed suggested that scarce CH resources and funding should be directed to effective health care services in more remote areas, because many urban areas already offer effective health care services (see quote in the next column). Additionally, government support and incentives, such as awarding funding based on CH performance measures, may be crucial to motivate or reward health care professionals and stakeholders.	“CH is essential in remote areas. It provides residents with access to health care services.”
32	Our interviewees expressed the importance of increasingly rapid innovation within the CH business model so that services can maximize the utility of current developments in CH-related technology, including remote sensing and rapid central analysis capabilities. The doctors expressing this view were primarily general practitioners with a business model and related funding, and their work mostly centered on patients who visited the general practitioners at a specific location for care (see quote in next column by a doctor working in a rural area).	“Although we seem to lack resources compared to urban areas, as long as we can offer unique value propositions, we can obtain essential resources from companies with CH.”
13	In some of these cases, stakeholders had cultural difficulties with CH—they argued that human contact between doctor and patient is irreplaceable and essential to health care. However, many of them did concede that contact may be flexible. These stakeholders assumed that CH is a business fad, rather than a necessary practice for patients. However, these views did not reflect the difficulties experienced in providing efficient and effective health care in remote regions.	“I have been doing medical services for a decade, and I support personal contact in health care; for me, connected health is just one of the formats of a business fad...”

Several industry interviewees seemed more concerned about the development of technology and its potential applications for CH than end users, based on their perceptions and their need for therapeutic care. This suggests more effective stakeholder interaction and business model innovation are required to coordinate end users’ needs and industrial provisions. Additionally, all industry interviewees were concerned about the sustainability of CH businesses beyond grant funding, and therefore emphasized the need to align technology development with a sustainable business model. These interviewees tended to use their connections and relationships to promote nascent supply chains for CH businesses that required more systematic development. In contrast, many academic interviewees considered CH as an emergent topic of innovation and cited a need for more interdisciplinary research and education beyond technological developments.

## Discussion

### Principal Findings

CH as a coexistent ecosystem includes all the stakeholders who work together to facilitate its implementation. Patients’ security and privacy are crucial concerns in the implementation process [36]. However, our interviewees indicated that their main priority is to test the feasibility of remote health care services, as all such services are required to follow current regulations concerning patients’ safety. This does not suggest that security and privacy issues can be compromised in exchange for ease of accessibility for health professionals and industry players [37]. On the contrary, following such standards is the precondition of practicing CH, even if some stakeholders think that there is no need to overemphasize the importance of following these standards. Below, we consider a few important issues and obstacles to implementing CH.

### Security Concerns

When asked about patients’ security concerns, some health professionals emphasized CH’s potential convenience and high quality of care. They often asserted that they believe that CH might be like online banking: offering services anytime, anywhere. In this study, governments bear the most responsibility for controlling the risks of using CH and for setting standards that will protect patients [38]. Although successful implementation of CH requires the cooperation of all stakeholders, only those who have power and impacts in the ecosystem can make final decisions regarding CH’s implementation. This explains why IT security professionals may not have the power to make decisions in the ecosystem, even though they play important roles.

Opinions regarding privacy and security differ. Some health professionals believe that patients’ and institutions’ security should be prioritized above the convenience of CH services, but others believe that efforts to make CH more feasible and effective should come first. In general, it is difficult to get all stakeholders to share common ground [39]. The power of decision making usually distinguishes the leaders in the ecosystem. This can be seen from the conversation between the government sectors and industry players. The latter expect government to form the standard of protocol, while the former insist on the respect of the free market.

### Interdisciplinary Talent

According to our interviewees, health professionals and governments have more power in the process of implementing CH than industry or patients. Industry usually follows government policies, as their products and services often require governments to initiate funding and infrastructure for the realization of these projects [40]. Patients’ needs are often interpreted through health professionals. Respondents indicated that interdisciplinary staff is crucial to enable the smooth operation of CH; however, it takes time to train and develop talents.

### Connected Health as a Platform to Facilitate Smart Health Care

Although many efforts have been made to improve the quality of health care, treating some diseases remains challenging because many caregivers and patients lack the information they need for effective treatment. Although CH provides a wide range of care delivery models, it can also be used simply to connect patients with the information they need for effective treatment [41]. In this way, CH provides a pathway to smart, precise health care. CH can put power back into the hands of patients [42], and the data collected from CH platforms can enable the provision of smart health care [43]. Through big data and artificial intelligence technologies, CH offers a platform that enables smart homes, smart cities, and patient-centered, personalized health interventions [44,45].

### Connected Health’s Innovative Strategies Based on Value Propositions

Scholars at Harvard Business School have discussed three innovation strategies that reflect how timely, smart, and targeted health care can be met through a CH platform. These strategies are the *infuse and augment* strategy, the *combine and transcend* strategy, and the *counteract and reaffirm* strategy [19].

The *infuse and augment* strategy involves the development of new services and products under the current service structure, without significant change to the attributes and functions of that structure. This strategy increases and emphasizes elements that deliver services that meet the needs and desires that are presently unfulfilled by major markets. A case in point is the Changhua Christian Hospital’s (CCH) eHealth and Diabetes Health Management Center, which was created in 2013 to meet the needs of an aging population with an increasing level of chronic health conditions. CCH had been a symbol of public welfare and social care for over a century, and many observers thought that the most plausible reaction to an aging society would have been to reduce the cost of care. However, the CCH believed that reducing the cost of care would have jeopardized the quality of care. Instead, CCH engaged with global institutions from developed countries, from which the trend of *aging in place* emerged:

For over a century, CCH has been playing the role of health care hub in the mid-south of Taiwan; now we are expanding our services and business to the global context.

Participant #55

Furthering this argument from the patient perspective, individuals prefer to remain in a familiar location, and may prefer to manage their own health rather than increase the number of medications they take. These insights explain why the CCH opted to create the aforementioned center instead of reducing the cost of care. Such a center offers more comprehensive and cost-effective services than conventional care. Creating the center allowed the CCH to avoid an across-the-board price reduction, in contrast to many hospitals that have responded to aging populations by reducing costs. In this case, the CCH viewed global market trends as opportunities for innovation and renewal:

We are proactive in absorbing new practices and exploring global trends...We discovered that our customers tend to follow the concept of “aging in place,” which echoes the global trend.

Participant #55

The head of the hospital is really supportive regarding both finances and resources in initiating this center so that we can offer a comprehensive service to our customers.

Participant #55

Taipei Medical University Hospital’s (TMUH) answer to an aging population’s call for seamless services represents another example of the *infuse and augment* strategy. One of the top hospitals in Taipei, TMUH introduced its telehealth-care service program in 2007, which offers consultation and over-the-phone care for patients outside standard working hours. This was achieved by integrating resources and increasing connected services. For example, customers who join the telehealth-care program are able to consult doctors online and book an appointment in advance. Connected devices, meal delivery, and laundry services are also available to ensure seamless and complete care. By augmenting its traditional health care services with these innovations, TMUH has thereby infused its value proposition with an online service:

Our clients can obtain access to health care information anytime they need to, even during out-of-office time.

Participant #34

We hope that our customers can use health care services in the future as if they are using online banking: 24 hours, seamless services.

Participant #34

The *combine and transcend* strategy offers a different, relatively radical approach to implementing CH. This strategy involves combining features of a service’s existing value propositions with quality-transferring changes arising from a trend. In this way, a novel experience is created, one which may lead the institution into a new market space. At first glance, employing resources to incorporate elements of a different domain into one’s core offerings sounds like it is not worthwhile. However, TaiDoc’s shift to integrate medical sensing solutions into its reputation for its integrated circuit design demonstrates how the *combine and transcend* strategy can be successful. In 2002, TaiDoc joined with hospitals to develop biosensing devices that can monitor patients’ health conditions remotely. By combining TaiDoc’s original value propositions for electronic supplies with those of health providers, the company entered a new field of engagement with the health sector. Within a decade, TaiDoc became the top manufacturer of blood glucose medical devices and now accounts for a significant share of the global market.

When the sales manager was asked why they are determined to jump into the business of medical devices, the response was as follows:

We are determined to expand our business to medical devices as our founder encountered an emerging need from his family.

Participant #31

As long as we saw the needs corresponding to the trend, we took the leap...although it was challenging, we enjoyed the fruits of success within 10 years.

Participant #45

Another example of the *combine and transcend* strategy is the case of Far EasTone Telecommunications. After seeing the potential of a smart health care industry, Far EasTone Telecommunications combined telecommunication services with medical devices to enable telehealth-care services. In order to make this *transcendence* of existing capabilities possible, they encouraged engineers to engage in work-related training and introduced their test models in their partnered hospitals. Far EasTone Telecommunications has now expanded their businesses to over 80 hospitals and institutions within the past 5 years and has become a leading telehealth services provider in Taiwan.

Our manager has an insightful vision of the market; she believes that telehealth-care will become a leading industry in the future.

Participant #18

Our boss considers a telehealth-care service to be a long-term strategy. He allows exploring of possibilities for a period of time and believes that success will come soon.

Participant #18

The *counteract and reaffirm* strategy entails understanding disagreements with the value statements of current services or products and reclaiming a new value proposition that is considered to be superior to existing values. An example of such a service is the Nanwui Foundation, a health and social care service established by a local physician from Tai-tong Health Center. Like other health facilities, they offer general health care services and health checks. However, the Nanwui Foundation also incorporates the fundamental needs of remote areas into its service manifesto; it holds educational workshops and has a green farm community, which allows residents to explore their values and elaborate on their talents. The case of the Nanwui Foundation supports the assertion that health care is not only about people’s physical health care needs, but also their need for dignity. Our interviewees reflected that patients are happier and healthier when they have a goal to pursue and a stage to play on:

When I saw these patients, they appeared old, poor, and weak, but when they participated in the social workshops that I organized, they became energetic and healthy.

Participant #57

Physical conditions are only a partial reflection of their health conditions...offering them a stage on which to perform that allows them to exert that their value may be more relevant to their well-being.

Participant #57

In remote areas, residents are poor and lack medical resources. When there is no Superman, we can be our own Superman.

Participant #57

### Connected Health’s Innovative Strategies

Although CH seems to provide promising solutions for the problems posed by an aging population with many chronic health conditions, these solutions are not easy or quick—innovative implementation strategies are required to enable its practices [46]. This is especially true because the players in the CH ecosystem compete for the same organizational resources and stakeholders. Therefore, each actor in the ecosystem needs to adopt different strategies to help them differentiate their value propositions and obtain useful resources [47]. Some interviewees indicated that many CH players failed due to lack of strategic planning. Therefore, this study adapted the strategies proposed by scholars to demonstrate how organizations use strategies to succeed with CH. These experiences may inspire players in various industries.

### Limitations and Future Work

This study explored how timely, smart, and targeted health care can be achieved through CH platforms. This means that some relevant content, such as a detailed stakeholder analysis, comprehensive consideration of security and privacy concerns, and detailed assessments of business models, was not described in a large amount of detail. These topics can be extended in future work to offer an inclusive picture of CH implementation. Apart from that, this study is based on the Taiwanese context and different cultural perspectives can be introduced and included in future studies.

### Conclusions

This study makes a relevant contribution to the literature by exploring perspectives on how trend-associated changes in customers’ opinions and behaviors influences health care and social care. Once these trends have been identified on a broad scale, appropriate innovation and intervention strategies can be developed and implemented. When innovation and entrepreneurship are correctly combined, CH businesses can be propelled and spectacular results can be expected. The *infuse and augment* strategy will permit CH businesses to emerge in successful and socially beneficial ways if the essential values of these businesses remain meaningful to consumers who engage with the service trends. If the results imply that the value statements are incongruent with consumers’ new expectations, it suggests that the current innovation strategy is insufficient and needs to be transcended or combined with another strategy to create a new value statement. If the current solutions are associated with negative impacts or conflict with the mainstream system, counteracting these aspects by reaffirming the core values of your business is an ideal strategy. The findings indicate that strategic planning could be beneficial to leverage resources for the implementation of CH. Moreover, continually revisiting business models and strategies is essential because of the rapid, ongoing technological changes across CH stakeholder groupings. In short, we believe that timely, smart, and targeted health care can be created through a CH platform.

## Appendix

Multimedia Appendix 1 Word frequency cloud of the interview data.

## Abbreviations

AMA: American Medical Association
BMI: Business Model Innovation
CCH: Changhua Christian Hospital
CH: connected health
ICT: information and communication technology
IT: information technology
TMUH: Taipei Medical University Hospital
