# Supplementary figures and images for: Smart, Remote, and Targeted Health Care Facilitation Through Connected Health: Qualitative Study
Source: J Med Internet Res. 2020 Apr 28;22(4):e14201. doi: 10.2196/14201 (PMC7218602; doi:10.2196/14201)

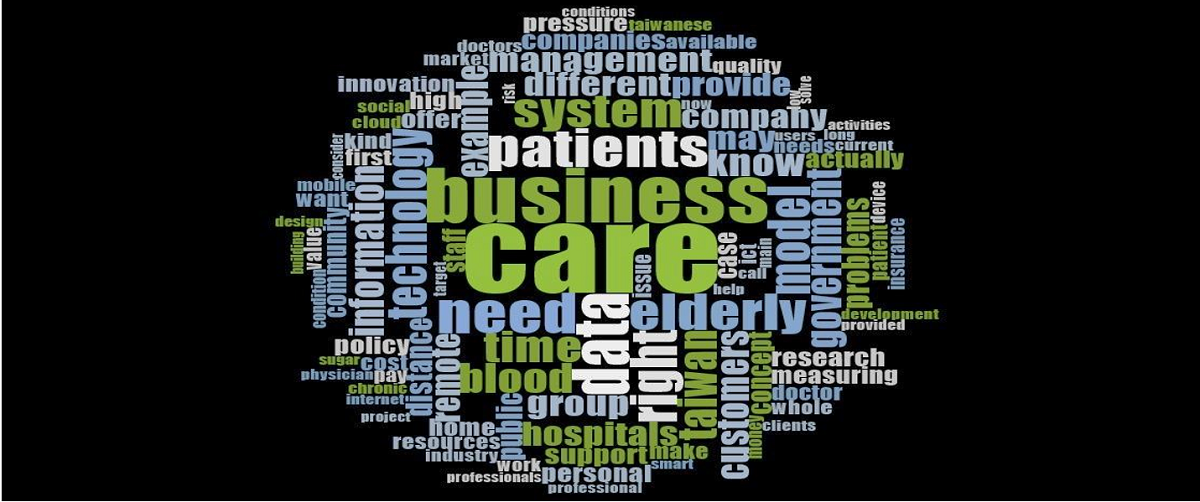

Supplement: Multimedia Appendix 1 [file jmir_v22i4e14201_app1.png]
